# Family psychoeducation for major depressive disorder – study protocol for a randomized controlled trial

**DOI:** 10.1186/s13063-016-1549-0

**Published:** 2016-08-30

**Authors:** Nina Timmerby, Stephen F. Austin, Kristian Ussing, Per Bech, Claudio Csillag

**Affiliations:** Psychiatric Research Unit, Mental Health Centre North Zealand, Copenhagen University Hospital, Dyrehavevej 48, Hillerød, 3400 Denmark

**Keywords:** Family psychoeducation, Major depressive disorder, Depression, Relatives, Relapse prevention, Expressed emotion

## Abstract

**Background:**

Major depressive disorder has been shown to affect many domains of family life including family functioning. Conversely, the influence of the family on the course of the depression, including the risk of relapse, is one reason for targeting the family in interventions. The few studies conducted within this area indicate that family psychoeducation as a supplement to traditional treatment can effectively reduce the risk of relapse in patients with major depression as well as being beneficial for the relatives involved. However, the evidence is currently limited. This study will investigate the effect of family psychoeducation compared to social support on the course of the illness in patients with major depressive disorder.

**Method/design:**

The study is designed as a dual center, two-armed, observer-blinded, randomized controlled trial. Relatives are randomized to participate in one of two conditions: either four sessions of manualized family psychoeducation or four sessions in a social support group led by a health care professional. Patients will not participate in the groups and will continue their treatment as usual. A total of 100 patients, each accompanied by one relative, will be recruited primarily from two outpatient clinics in the Capital Region of Denmark.

The primary outcome is the occurrence of depressive relapse at 9-month follow-up defined as a score ≥7 on the Hamilton six-item subscale. Secondary outcomes will include time to relapse.

**Discussion:**

It is hoped that the results from this study will help to clarify the mechanisms behind any beneficial changes due to family psychoeducation and provide information on the long-term effect of this intervention for both patient and relatives. If the results are positive, the family psychoeducation program may be suitable for implementation within a clinical setting.

**Trial registration:**

ClinicalTrials.gov Identifier: NCT02348827, registered 5 January 2015.

## Background

Depression is a frequently occurring disease with an estimated prevalence in Denmark of 3–4 % [1] consistent with findings in other Western European countries [2].

The risk of relapse and recurrence is high [3]. Patients having experienced their first depressive episode have a more than 50 % risk of suffering from a new episode [4]. Furthermore, the risk of subsequent recurrence increases with the number of previous depressive episodes [5].

A robust relationship has been established between family factors and the course of depression, including the risk of relapse [6]. More specifically, relapse has been found to be predicted by the level of expressed emotion (EE) in key relatives defined as a critical, hostile or emotionally over-involved attitude or behavior toward the patient [7]. Several studies have confirmed that patients living with high-EE relatives have a significantly higher risk of relapse than patients living with low-EE relatives [8–11].

Conversely, depression affects the function of the family, as well as impacting on the life of the relatives. Viewing the family as whole, families of patients suffering from depression are characterized by reduced family functioning compared to both nonclinical families and families of patients with schizophrenia or bipolar disorder [12]. Furthermore, relatives of depressed patients report significantly lower levels of quality of life in terms of “psychological wellbeing” compared to the general population [13].

Depression not only causes distress for the patient but also for their close relatives. Relatives of patients with depression report significantly more difficulties than nonclinical families and these difficulties seem to persist even when there is symptomatic remission [14].

Thus, there is a clear link between family functioning and the course of depression. It has previously been suggested that family intervention would be a meaningful and important supplement to psychotherapy and psychopharmacological treatment [15]. These family-oriented practices include a spectrum of therapeutic interventions, covered by the term family therapy and also include family psychoeducation (FPE) [16]. Family psychoeducation is already an evidence-based practice in the treatment of both schizophrenia and bipolar disorder [17].

Relatively few trials have investigated the effect of FPE on major depression [18]. One recent randomized trial found that if relatives received a brief intervention consisting of FPE, the patients who had achieved full or partial remission from an acute depressive episode had a significantly lower relapse rate in a 9-month follow-up period compared to the control condition. However, the comparison was between FPE and treatment as usual (TAU) where the TAU control group provided no intervention for the relatives. Thus, it was not possible to ascertain whether the significant beneficial effect of FPE was due to nonspecific factors (e.g., support from the group) or to active psychoeducation [19].

Another open study investigated the effect of a similar FPE program on the psychosocial burden experienced by the relatives of patients with major depression [20]. This study found that relatives participating in four sessions of multi-FPE had a significant improvement with regard to care burden, EE and mental health. However, no control group was included. Furthermore, no evaluation of patient-related outcomes was undertaken, relatives were only evaluated post intervention and no information on the long-term consequences of the intervention was collected.

Current results on the effect of FPE as a supplement to psychotherapy and psychopharmacological treatment for major depression seem promising and may be effective in reducing the risk of depressive relapse. However, the evidence is still limited [19]. Due to shortcomings in the design of this trial it was not possible to identify the mechanisms behind change. Furthermore, the results from this study did not clearly support the hypothesis that the treatment effect was mediated by a reduction of expressed emotion (EE) [19]. Expressed emotion was low at baseline and since the study was conducted in Japan it is reasonable to assume that levels of EE may vary across cultures. Previous studies have found EE to be low in Japanese settings [21].

Additionally, little is known about the effect of FPE in depression on relative-related outcomes, such as quality of life. Quality of life is an important aspect to investigate since the relatives of patients with depression report considerable distress and are at risk of developing depression themselves [22]. Family psychoeducation may improve emotional distress and care burden experienced by the relatives [20] but further research is needed. Family psychoeducation is also in line with the concept of “positive psychiatry,” a branch within psychiatry that is concerned with interventions promoting wellbeing [23, 24].

In summary, despite some studies highlighting the potential benefits of FPE in major depression there is a need to conduct more rigorous trials that include an active control group to help to identify the mechanisms behind any benefits derived from FPE. New studies also need to examine the potential long-term benefits of FPE for patients and relatives and the broader impact of FPE on psychological constructs such as wellbeing and quality of life.

### Aim

The aim of the present study is to compare an intervention consisting of FPE to an active control intervention of social support for relatives of patients with a diagnosis of major depression.

The following hypotheses are proposed:

1. Psychoeducational intervention for relatives will reduce the risk of depressive relapse (defined as a score on The Hamilton six-item subscale, HAM-D_6_ ≥ 7), among remitted depressed patients (HAM-D_6_ < 7) compared to the active control condition
2. Psychoeducational intervention for relatives will reduce the time to achieve full symptomatic remission (defined as HAM-D_6_ < 5) among partially remitted depressed patients (HAM-D_6_ = 8–12) compared to the active control condition
3. Psychoeducational intervention for relatives will reduce depressive symptoms (measured on the HAM-D_6_) among currently depressed patients (HAM-D_6_ ≥ 13), compared to the active control condition

#### Secondary aims

The study has as the secondary goal of exploring any differences between the two groups on family functioning, wellbeing, level of disability and EE for patients and relatives.

## Method

### Design

The study is a dual center, two-armed, observer-blinded, randomized controlled trial investigating the effect of FPE compared to an active control group on the course of the illness in patients with a diagnosis of major depression.

The study is divided into two phases, an intervention phase and a follow-up phase. During the first phase the relatives will, after inclusion and baseline assessments, be randomised to one of two conditions comprised of four weekly sessions, of either a FPE program (intervention group) or social support for relatives (active control group).

The second phase is a follow-up conducted 9 months after completion of treatment involving the assessment of both patients and relatives.

The 9-month follow-up period was selected as a previous study has found a significant effect of FPE, reducing the risk of relapse within that timeframe [19]. Furthermore, this timeframe allowed for the collection, analysis and reporting of results within the course of a 3-year PhD project.

All patients during the study period will continue their TAU at the outpatient clinic or with their private psychiatrist. Treatment decisions will not be affected by the patients’ decision to participate or not in the study and there will be no restrictions regarding psychopharmacological treatment. Information about psychopharmacological treatment will be registered at both baseline and endpoint in order to document any changes.

### Participants

Study participants include both patients and relatives. Each patient in the study will be accompanied by one relative.

Inclusion criteria for the patients are: (1) age between 18 and 75 years, (2) a diagnosis of “major depressive disorder”, either single-episode or recurrent depression according to the *International Classification of Diseases-10* (ICD-10), established by a certified psychiatrist and verified by the MINI International Neuropsychiatric Interview (MINI) [25], and (3) living together or in regular contact with an adult (over 18 years of age) relative who the patient considers as emotionally important and who is available for intervention.

Regarding hypotheses 1 and 2, patients will be included if they are in remission or partial remission at the inclusion time defined as a score <13 on the Hamilton Rating Scale for Depression (HAM-D_17_) [26].

We define remission as a score on the HAM-D_17_ ≤ 7 as generally accepted [27] and partial remission as where the patient no longer meets the diagnostic criteria but more than minimal symptoms persist [28].

We consider a HAM-D_17_ score from 8–2 to be compatible with the definition “more than minimal symptoms” and will, therefore, define partial remission as a score on the HAM-D_17_ from 8–12.

Regarding the test of hypothesis 3, patients will be included if they are fully symptomatic defined as a HAM-D_17_ score ≥13 at the time of the inclusion.

Exclusion criteria for patients are: insufficient knowledge of the Danish language, diagnosis/clinical signs of dementia, alcohol, drug or medicine abuse, psychotic symptoms, comorbidity of severe personality disorder, having undergone electroconvulsive therapy (ECT) treatment during the index depressive episode, duration of the current depressive episode exceeding 2 years, i.e., chronic major depression, and duration of the period with stable remission exceeding 3 months (as regards the patients with a HAM-D_17_ score <7 at the time of inclusion).

Inclusion criterion for relatives is being aged between 18 and 75 years. In order to reflect the clinical setting and maximize generalizability of study findings, no other exclusion criteria for relatives will be enforced apart from insufficient knowledge of the Danish language.

### Recruitment

Patients will be recruited at the outpatient clinics from two centers affiliated to the University of Copenhagen: the Mental Health Centre North Zealand, in Hilleroed, and the Mental Health Centre Copenhagen, as well as from private psychiatrists in the Capital Region.

The investigator will evaluate whether the criteria for inclusion are met and the ICD-10 diagnosis of depression will be verified by means of the MINI International Neuropsychiatric Interview [25]. After baseline assessments the relatives are randomly allocated to one of the two groups as described in the following paragraph.

### Randomization

After baseline assessments the relatives are randomly assigned (1:1) to either the psychoeducation group or the social support group. The randomization design is a blocked design with 10 relatives in each block. In order to ensure a balanced distribution of both “treatments” in each of the two blocks, a computerized randomization procedure (Microsoft Excel©) will be used to generate randomization codes. The codes will be placed in envelopes. The randomization performed each time a relative is included in the study is performed by a secretary affiliated to the Research Unit at Mental Health Centre North Zealand but not otherwise involved in the study.

#### Blinding

Assessments of both patients and relatives during the study period will be conducted by the investigator who is blinded to the group allocation of the relatives.

### Treatment

#### Psychoeducation group

Relatives assigned to this group will participate in a manualized psychoeducation program developed by the authors. The psychoeducation program is partially based on the McFarlane model for multifamily groups [29] as well as on an intervention tested in a similar way applied in a Japanese study [19].

The program will consist of a total of four sessions, each with a duration of 2 hours, in comparison to six sessions in the McFarlane Multifamily Treatment Program for major depressive disorder [29]. The decision to have four sessions of psychoeducation was based on a study that demonstrated that this treatment length was associated with reducing the risk of new depressive episodes [19]. Furthermore, feedback from the pilot study (see below) indicated that patients and relatives found four sessions to be the optimal treatment length.

In contrast to the McFarlane program, patients will not participate in the psychoeducation sessions. This slight alteration in treatment format was based on the previous studies that had shown that FPE interventions that involved relatives only could also be effective in helping people with depression [30–32] and had the extra advantage of not burdening the patient [19].

All groups will be led by one to two experienced psychiatric nurses who are highly trained in mood disorders, and regularly conduct structured psychoeducation programs for patients and their family members. Each group will consist of five relatives and the sessions will be carried out weekly for four consecutive weeks.

A pilot study consisting of 10 patients and their respective relatives was conducted to in order to test the feasibility and clinical applicability of the psychoeducation program. The program was amended based on feedback from both group leaders and the participating relatives.

Each of the four sessions have the same structure and are divided into 35 min of psychoeducation based on transmission of knowledge about depression and the concept of EE, followed by 70 min of problem-solving exercises based on problem areas proposed by the relatives. The psychoeducation program consists of four themes covered in each session: causes and symptoms of depression, treatment of depression, communication and prevention of new depressive episodes. In the first session both group leaders and participating relatives will introduce themselves to the group and group rules concerning confidentiality and respect will be emphasized. For each of the four sessions specific topics will be covered including: symptoms of depression, how the diagnosis is established, the psychosocial model as an approach to mental illness, etiological factors and stressors, antidepressive medication and side effects, psychotherapy, the importance of maintenance treatment, how depression can affect communication in the family in a negative way, how the roles and responsibilities of the family members can change because of depression, the concept of EE and how critical, hostile attitudes and overinvolvement can affect the patient negatively, desirable ways of communicating in the family, the risk of new depressive episodes, early symptoms of depression, coping strategies of relatives and how to take care of oneself and where to seek additional information and help as a relative.

The problem-solving exercises are designed to provide participants with problem-solving skills focusing on approaches to cope with situations in the family, including situations characterized by a high degree of EE and are a way for the relatives to apply the knowledge obtained in group sessions to their personal lives. The problem-solving approach is the same as that used by McFarlane [29]. In every session the exercise begins with a round where all the relatives can bring forward a problem experienced by themselves as a relative to a person with depression. Afterwards the group leaders will help the group to select one of the problems to be solved. The following steps include: (1) definition of the problem, (2) listing of possible solutions proposed by the group members by means of brainstorming, (3) discussion of the advantages and disadvantages of the solutions proposed, (4) the group member who introduced the problem chooses the best suggested solution, and finally (5) that person reflects on how the solution can be carried out. In the following session the group member is furthermore requested to give feedback on how the implementation went [29].

The structure of the problem-solving model application has been used in several therapeutic programs and is thought to be well-suited for use in a healthy population, in this case relatives, because of its educational approach [33].

The roles of the group leaders include providing oral information to the relatives on the topics listed previously as well as leading the relatives through the problem-solving exercise. As described by McFarlane [29] one of the group leaders will guide the participants through the steps of the problem-solving exercise while the other group leader will write down the steps and supplement them with additional solutions. Group leaders would agree prior to the session on the division of roles.

The material for the use of the psychoeducation group consists of a manual for the group leaders as well as handouts for the relatives summarizing the topic of the day and is handed out at the end of each session. Each of the problem-solving exercises is based on four fictive cases describing common situations that can be experienced by relatives of people suffering from major depression. Furthermore, a working sheet for the problem-solving exercises originating from the McFarlane program is applied in the sessions [29].

#### Social support group

Our study design will provide a matched active control condition where relatives in the social support group will attend the same number of sessions of the same duration as relatives in the intervention group. A support group is defined as a network of people who can share a common experience [34].

Each group will be led by one professional expert (psychologist trainee, psychiatric nurse or psychologist). The group leader is only present in order to “facilitate” the discussion of the relatives. The relatives will be informed that the purpose of the group meetings is to provide them with a forum where they can meet and exchange experiences with like-minded relatives. The relatives are also informed that the group leader is not present at group meetings in the role of expert and will, therefore, not provide any psychoeducational intervention. Regarding the content of the session, the participating relatives themselves decide which topics they find relevant and want to bring up. At the end of each session the group leader will document in writing which topics the group has the touched on during the session.

This type of group with a professional group leader proving a minimum of intervention has previously been used as an active control when testing the effect of psychoeducation in a randomized controlled trial [35].

#### Fundamental differences between the FPE group and the active social support group

The primary difference between the two groups is that the FPE group provides the relatives with specific knowledge about important topics within major depression as well as providing active strategies when working with problems identified by the group members. The FPE group is provided in a structured format in order to ensure that all relevant topics and problem-solving strategies are covered. The content of the active support group is “open” and determined by group members, not the facilitator. Whilst there may be some overlap in the topics discussed within these two groups, the facilitator in the active control group does not provide any structured information or strategies to participants in response to the issues raised by group members.

It is acknowledged that mutual support groups can have a positive effect on outcomes for patients and the relatives. However, we hypothesize that any positive effect found in our support group will disappear when the intervention ceases. Numerous studies have shown that benefits from support groups are often short-term [34]. Conversely, we postulate that any positive effect found in the FPE group will be sustained after the intervention ends, since this intervention teaches the relatives strategies that can be used to address problems and improve the relationship with their relative. Thus, FPE has a developmental focus where relatives can implement new information and learned strategies over a period of time, which in turn has the potential to positively influence the relationship between the relative and the person with depression. This treatment effect of FPE, which develops over time, is reflected in the primary outcome of the study: occurrence of relapse at 9-month follow-up rather than change post intervention.

### Fidelity and quality assurance

The psychoeducation group will be instructed by psychiatric nurses who already have extensive experience in performing manualized psychoeducation. Furthermore, the group leaders of both the FPE group and the control group obtained training in the use of the respective manuals during the pilot study. The training and supervision during the pilot study was performed by one of the authors (SFA) who is a specialist in psychotherapy and an experienced certified supervisor.

All sessions in the both the intervention group and the control group will be videotaped. To examine the treatment fidelity in the intervention group, an independent assessor will use a checklist based on the specific content of the treatment manual to evaluate the video recordings in a random sample of taped sessions to ensure that all key elements of each session are carried out. Likewise, a random sample of sessions taped in the control group will be viewed and evaluated by an independent assessor in order to ensure that the facilitator role of the group leader is carried out neutrally and that no structured elements of FPE are included.

Rating and evaluation of the speech samples from The Five Minute Speech Sample (FMSS) (see below) will be carried out by two of the authors (NT and SFA) by use of the standard FMSS coding system [36]. The authors NT and SFA have already received extensive certified training and have reached excellent agreement (97 %) with the criterion coder and developer of the coding system [36].

Both of the raters of the FMSS speech samples will be blinded to group allocation of the relatives.

### Assessments

Both patients and relatives will be assessed multiple times during the study period. Briefly, assessments are scheduled at (1) baseline, where sociodemographic data of both patients and relatives as well information on illness history and psychopharmacological treatment will be obtained, (2) within 2 weeks after the relatives have attend the fourth and last group session, and (3) at 9-month follow-up. A flow diagram for assessments during the study period is shown in Table 1.

**Table 1:**
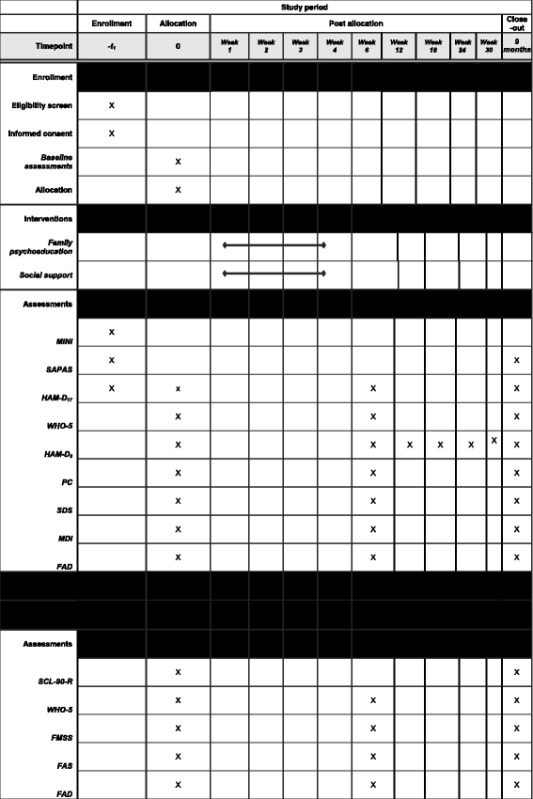
Flow diagram for enrollment, interventions and data collection at baseline and follow-up

*FAD* The Family Assessment Device (36-items), *FAS* The Family Attitude Scale, *FMSS* The Five Minute Speech Sample, *HAM-D*_*6*_ Hamilton six-item subscale, *MINI* MINI International Neuropsychiatric Interview, *PC* Perceived Criticism Scale, *SAPAS* The Standardized Assessment of Personality-Abbreviated Scale, *SCL-90-R* The Symptom Checklist-90-Revised, *SDS* The Sheehan Disability Scale, *WHO-5* The World Health Organization Well Being Index

Furthermore, telephone calls will be made to patients every 6 weeks in the follow-up period in order to obtain information on the presence of depressive symptoms measured by the HAM-D_6_.

Patients will be told to contact the principal investigator when recognizing a worsening of depressive symptoms. Furthermore, the patients’ medical records will be screened in order to identify information on relapse. Both the relatives and private psychiatrists have the opportunity to contact the principal investigator if they suspect a depressive relapse.

### Measures

The diagnosis of major depressive disorder is verified by use of the MINI. The MINI will also be applied in order to screen for the presence of axis-I comorbid psychiatric disorders according to the *Diagnostic and Statistical Manual of Mental Disorders, 4th Edition* (DSM-IV). The MINI is a short, structured diagnostic interview, with an administration time of approximately 15 min, that assesses the most important axis-I diagnoses, according to the DSM-IV and the ICD-10 [25].

Furthermore, patients are screened for DSM-IV axis-II disorders using The Standardized Assessment of Personality-Abbreviated Scale (SAPAS), a structured interview consisting of eight items [37] which has been shown to have adequate clinical applicability in patients with a primary diagnosis of depression [38].

Patient-reported outcomes include the following measures: the Major Depression Inventory (MDI) is a questionnaire containing 10 depressive symptoms from both the ICD-10 and the DSM-IV, with a specific timeframe of the previous 2 weeks. Each item is scored from 0 to 5, and the 10 items are summed to a total score. The MDI can be used as either a rating scale for assessment of the degree of depressive symptoms *or* as a diagnostic tool [39] with cutoff scores for mild, moderate and severe depression, respectively [40]. The Hamilton six-item subscale (HAM-D_6_) is a self-rating scale covering the core symptoms of the HAM-D_17_ [41]. The HAM-D6 has high clinical validity [42] as well as adequate psychometric validity [43]. The World Health Organization Well Being Index (WHO-5) consists of five questions and is one of the most used questionnaires in the measurement of subjective psychological wellbeing [44]. The Sheehan Disability Scale (SDS) is a three-item self-report measure of disability and impairment within the domains of work, social life and family life [45]. The Perceived Criticism Scale (PC) is four-item scale used to assess how critical the patients consider themselves to be of their relative and how critical the patients consider the relative to be of them [10].

Assessment instruments filled out by the relatives include the WHO-5 as well as the following:

The Symptom Checklist-90-Revised (SCL-90-R) consists of 90 items and assesses symptoms of psychopathology within nine different symptom dimensions [46].

Expressed emotion (EE) is measured by The Family Attitude Scale (FAS) [47] and The Five Minute Speech Sample (FMSS) [36]. The FAS is a measure of EE rated by the relatives themselves. It consists of 30 items and assesses self-reported critical and hostile attitudes towards the patient.

In the FMSS the relative is instructed by an interviewer to talk about their thoughts and feelings regarding the patient. The relative is told to speak for 5 min without the interviewer asking questions or otherwise interrupting. The speech sample is recorded and later coded by a trained rater in order to classify the relative as either “High EE” or “Low EE.”

The FMSS is an alternative to the very time consuming “gold standard” way to measure EE, the Camberwell Family Interview (CFI) [48]. FMSS has been found to be a feasible method to evaluate EE in families of patients with mood disorders [49]; predictive validity with regard to the clinical outcome of depression has also been demonstrated [50].

Furthermore, a modified version of The Family Assessment Device (FAD) [51] will be used. The modified version consists of 36 items and two subscales measuring wellbeing and illbeing aspects of family functioning, respectively [52]. This new version of the FAD will, furthermore, be psychometrically validated as a part of the current study.

In order to quantify the relatives’ experience with participation in the groups, they will after the last group session fill out a modified version of the WHO treatment satisfaction questionnaire [53].

### Outcomes

#### Primary outcome

The primary outcome is occurrence of depressive relapse defined as a score ≥7 on the HAM-D_6_ [54] in the 9-month follow-up period (hypothesis 1) among remitted patients.

#### Secondary outcomes

Secondary outcomes are time to relapse, defined as the number of weeks from baseline to relapse; time to full remission defined as a HAM-D_6_ score <5 [54] among the partially remitted patients (hypothesis 2); and the reduction of depressive symptoms in the HAM-D_6_-score (hypothesis 3).

A number of exploratory questions will be examined in regard to the impact of the two interventions: for patients’ depressive symptoms (MDI), subjective wellbeing (WHO-5), disability (SDS), family functioning (FAD) and perceived criticism (PC), and for the relatives: subjective wellbeing (WHO-5), family functioning (FAD) and EE (FAS and FMSS).

### Statistics

#### Sample size calculation

Regarding the testing of hypothesis 1, only one similar study in the literature exists that tests an invention similar to the one applied in our study. Shimazu et al. [19] here found a relapse rate in the 9-month follow-up period of 8 % in the intervention group and 50 % in the control group. Based on these findings, 21 patients should be included in each group when using a power of 80 % and type-I error probability associated with the test of the null hypothesis of 5 %. However, we wish to make a more conservative estimate since we acknowledge that our active social support group may also have a positive impact on the outcome results. We therefore plan to include 30 patients in each group to the test hypothesis 1.

Regarding the testing of hypotheses 2 and 3, there are to the best of our knowledge no studies describing this type of intervention to this specific patient group. Therefore, a sample size calculation cannot be performed. Consequently the testing of hypotheses 2 and 3 is more exploratory and we plan to include 15–20 patients in each group to examine both these hypotheses.

#### Statistical analyses

For the statistical analysis SAS software ® will be used.

Baseline characteristics of the invention group and the control group, respectively, will be compared using a *χ*^2^-test regarding categorical variables and an independent samples *t* test regarding numerical variables. The comparison of baseline characteristics will be performed for the whole group of patients independent of subgroup affiliation based on depression levels at baseline.

The number of recurrences in the two groups will be compared using the Mann-Whitney *U* test.

Time to relapse as well as time to full remission (for patients only partially remitted at baseline) will be analyzed using the Kaplan-Meier survival analysis.

To analyze whether the degree of EE in relatives at baseline will be associated with poorer outcome, Cox proportional hazard regression analysis will be applied.

Intention-to-treat analyses will be carried out, meaning that analyses will include all subjects who are randomized.

Regarding missing data the method of Last Observation Carried Forward (LOCF) will be applied.

## Discussion

To our knowledge this is the first randomized controlled trial comparing Family Psychoeducation (FPE) with an active control group in order to clarify the mechanism behind any observed change due to FPE as well as examining the long-term effect of this intervention for patients and relatives.

It is hoped that the intervention will impact positively on both patients and relatives by improving family functioning. Specifically, family psychoeducation is designed to provide the relatives with better strategies in order to cope and support depressed family members, thereby facilitating a quicker response to treatment. The knowledge and new strategies can not only help relatives to recognize depressive symptoms in, but also help them provide better support to, the depressed person and potentially prevent a depressive relapse and help to maintain recovery.

The intervention represents a holistic approach to treating depression by recognizing that depression not only affects the individual suffering from depression but also their wider social network. This relationship is two-way where poor family functioning could increase the risk of relapse whilst good family functioning could be a protective factor against depressive relapse. If this intervention reduces the risk of depressive relapse and promotes recovery it could also have a broader impact of decreasing the burden of disease. Systematic evaluation of the effect of FPE fills an important gap in current research by proposing the patient-relative relationship as a potential mechanism to influence outcome in depression.

The trial includes a heterogeneous group of patients with depression recruited from diverse geographically situated outpatient clinics as well as from private psychiatrists. Hence, it is hoped that results from this study will be highly generalizable to clinical practice. Implementation into daily practice can be further facilitated by the fact that the intervention is not expensive or complex.

## Trial status

The first relatives were randomized in April 2015. The inclusion of participants is ongoing and is expected to continue until mid-2016.

## Abbreviations

CFI: The Camberwell family interview
EE: Expressed emotion
FAD: The Family Assessment Device
FAS: The Family Attitude Scale
FMSS: The Five Minute Speech Sample
HAM-D_6_: Hamilton six-item subscale
MINI: MINI International Neuropsychiatric Interview
PC: Perceived Criticism Scale
SAPAS: The Standardized Assessment of Personality-Abbreviated Scale
SCL-90-R: The Symptom Checklist-90-Revised
SDS: The Sheehan Disability Scale
WHO-5: The World Health Organization Well Being Index
